# Lectin array and glycogene expression analyses of ovarian cancer cell line A2780 and its cisplatin-resistant derivate cell line A2780-cp

**DOI:** 10.1186/s12014-017-9155-z

**Published:** 2017-05-23

**Authors:** Ran Zhao, Wenjun Qin, Ruihuan Qin, Jing Han, Can Li, Yisheng Wang, Congjian Xu

**Affiliations:** 1 Institute of Biomedical Sciences, Fudan University, 138 Yi-Xueyuan Road, Shanghai, 200032 People’s Republic of China; 2 Key Laboratory of Glycoconjugate Research Ministry of Public Health, Department of Biochemistry and Molecular Biology, School of Basic Medical Sciences, Fudan University, 138 Yi-Xueyuan Road, Shanghai, 200032 People’s Republic of China; 3 Obstetrics and Gynecology Hospital of Fudan University, 419 Fang-Xie Road, Shanghai, 200011 People’s Republic of China; 4 Department of Obstetrics and Gynecology of Shanghai Medical School, Fudan University, Shanghai, 200032 People’s Republic of China; 5 Shanghai Key Laboratory of Female Reproductive Endocrine Related Diseases, Shanghai, 200032 People’s Republic of China

**Keywords:** Ovarian cancer, Drug resistance, Glycomics, Gene expression analysis

## Abstract

**Background:**

Ovarian cancer is one of the most lethal gynecological malignancies, in which platinum resistance is a common cause of its relapse and death. Glycosylation has been reported to be involved in drug resistance, and glycomic analyses of ovarian cancer may improve our understanding of the mechanisms underlying cancer cell drug resistance and provide potential biomarkers and therapeutic targets.

**Methods:**

The serous ovarian cancer cell line A2780 and its platinum-resistant counterpart A2780-cp were used in this study. We performed a lectin array analysis to compare the glycosylation patterns of the two cell lines, a gene expression array was employed to probe the differences in glycogenes. Furthermore, the results were verified by lectin blots.

**Results:**

A2780-cp cell exhibited stronger intensities of *Lens culinaris* (LCA) *Canavalia ensiformis* (ConA), and *Lycopersicon esculentum* (LEL) and weaker intensities of *Sambucus nigra* (SNA) lectins. The gene expression array analysis revealed increased expression of Fut8, B3gnt4, B3gnt5, B4galt2 and decreased expression of Fut1 and ST6GalNAc 6 expression were evident in the A2780-cp cells. The lectin blot confirmed the differences in LCA, ConA, SNA and LEL between the A2780 and A2780-cp cells.

**Conclusions:**

The combination of the lectin and gene expression analyses showed that the levels of core fucosylation and poly-LacNAc were increased in the A2780-cp cells and the levels of Fuc α1-2(gal β1-4) GlcNAc and α2-6-linked sialic structures were decreased in the A2780-cp cells. These glycans represent potential biomarkers and might be involved in the mechanism of drug resistance in ovarian cancer.

**Electronic supplementary material:**

The online version of this article (doi:10.1186/s12014-017-9155-z) contains supplementary material, which is available to authorized users.

## Background

Ovarian cancer is one of the most lethal gynecological malignancies [1], because of the difficulty of early diagnosis and its recurrence, accompanied by drug resistance [2–4]. Although 80% of patients are sensitive to the initial treatment for ovarian cancer, including optimal cytoreductive surgery and subsequent platinum-based chemotherapy, his disease is ultimately lethal in 70% of patients because of relapse and drug resistance [5]. Thus, new treatments to overcome the recurrence and drug resistance of ovarian cancer are still urgently needed.

Glycosylation is the most prevalent post-transcription modification, and participates in many physiological and pathological processes. Proteins exhibit aberrant glycosylation in cancer [6, 7], and Glycan has been shown to play an important role in drug resistance of cancer. First, the altered glycosylation of transporter proteins results in altered sensitivity to chemotherapy drugs. For instance, multidrug resistance phenotype can be reversed by inhibiting the glycosylation of transporter proteins (P-glycoprotein) in ovarian cancer cells [8]. ATP binding cassette transporters in ovarian carcinoma cells that are resistant to oxaliplatin show defective glycosylation and increased expression levels [9]. Second, variations in glycosylation are involved in some signaling pathways that are critical for drug resistance mechanisms, such as the phosphoinositide 3 kinase (PI3 K)/Akt pathway [10–13]. In addition, glycomic analyses can detect biomarkers for the early diagnosis of cancer [14, 15], and the biomarkers can serve as prognostic indicators [16], or aid in the discrimination of resistant forms [17]. Thus, glycan can be used for precisely targeting combined with traditional chemotherapy, and it can be used to determine the most suitable chemotherapy drug prior to treatment.

However, few researches have studied on glycomic analyses of drug-resistant ovarian cancer, with majority of studies focusing on serum samples [17, 18]. Because 90% of ovarian cancers are of epithelial cell origin [19], cancer cells are more stable than plasma sample, and more convenient for mechanistic research. Thus, glycosylation profiling of cells can be easily applied in further research. The A2780 cell line and its cisplatin subline A2780-cp are recognized cell models that are used to investigate the mechanism of cisplatin resistance. In this study, we provide an overview of glycan and glycogenes expression in the A2780 and A2780-cp cell lines using a lectin array and a human gene expression array to identify potential biomarkers or targets and provide information on the involvement of glycan in the cisplatin resistance mechanism.

## Methods

### Cell culture

The high-grade ovarian cancer cell line A2780 and its cisplatin resistant subline A2780-cp were obtained from the Obstetrics and Gynecology Hospital of Fudan University (China) and grown in DMEM (Gibco Life Technologies, Carlsbad, CA, USA) supplemented with 10% fetal bovine serum (Gibco) and 100 U/ml penicillin/streptomycin (Gibco) at 37 °C in a humidified atmosphere of 5% CO_2_.

### CCK8 assay

Cell growth and viability were measured using the CCK8 assay (Dojindo, Kumamoto, Japan). Briefly, the A2780 and A2780-cp cells were plated in 96-well plates (5000/well). After 12 h, the cells were treated with various concentrations of cisplatin (0, 10, 20, 30, 40, and 50 μg/ml) for 24 h, and 10 μl of the CCK8 reagent was subsequently added to the cells. The optical density (OD) value of the cells was then measured at 450 nm using an EPOCH ELISA reader (BioTek Instruments, Vermont, USA) according to the manufacturer’s instructions.

### Lectin microarray

The protocol used for lectin microarray analysis was previously described [20]. Briefly, A2780 and A2780-cp cells were collected and washed three times with PBS, and 1 × 10^5^ cells were then suspended in 1 ml of PBS-T (containing 1% Triton X-100), followed by sonication for 15 min. Next, the samples were centrifuged at 14,000*g* for 15 min, and the supernatant was recovered. After protein quantification with a Micro BCA Assay Kit (Thermo Scientific), the proteins were labelled with the fluorescent dye Cy3 (Thermo Scientific). The linear range of standard glycoprotein (Immunoglobulin G) for this LecChip (Glyco Technica) was 10–200 ng/ml. A dilution series from 2 μg/ml down to 31.25 ng/ml (seven levels), were recommend to take binding curves. Our samples were applied to a LecChip at a concentration of 500 ng/ml, followed by incubation at 20 °C for 16 h. The chip was then scanned with a GlycoStation Reader 1200 confocal scanner (Glyco Technica). For normalization, the intensity of each well was divided by the mean intensity of the chip from 135 wells in total (45 lectins with three replicates). We repeated the lectin microarray analysis using independent samples to overcome any biological biases. Lectins that showed significant change in same direction in the two independent lectin array experiments were considered as significantly differentially expressed lectins. All lectins information was shown in Additional file 1.

### Total RNA extraction and gene expression microarray

The total RNA was extracted using TRIzol™ reagent (Invitrogen, Carlsbad, CA, USA) and quantified with a NanoDrop ND-2000 spectrophotometer (Thermo Scientific). The RNA integrity was assessed using an Agilent Bioanalyzer 2100 (Agilent Technologies). The sample labeling, microarray hybridization and washing were performed based on the manufacturer’s standard protocols. Briefly, the total RNA was transcribed to obtain double-stranded cDNA, followed by cRNA synthesis and labeling with Cyanine-3-CTP. The labeled cRNAs were hybridized onto the microarray [Agilent SurePrint G3 Human Gene Expression v2 (8*60 K, Design ID: 039494)]. After washing, the arrays were scanned with an Agilent Scanner G2505C (Agilent Technologies).

### Quantitative real-time PCR analysis

 Total RNA was extracted with the TRIzol™ reagent (Invitrogen, Carlsbad, CA, USA), and 2 μg of RNA was used to reverse transcription using an RT Master Mix kit (Takara, Shiga, Japan). An ABI 7500 Fast Real-time PCR system (Applied Biosystems, Switzerland) were used for Real-time PCR analysis. A 2-μl aliquot of cDNA was mixed in 20 μl system using the SYBR-green Premix Real-time PCR kit (Takara) system according to the manufacturer’s instruction and amplified for 40 cycles (15 s at 95 °C, 30 s at 60 °C). The primer sequences were as shown in Additional file 2.

### Lectin blot

The total proteins isolated from the A2780 cells and A2780-cp cells were analyzed via SDS-PAGE and lectin blotting. Briefly, the samples were separated via 10% SDS-PAGE and transferred to polyvinylidene difluoride (PVDF) membranes (Millipore, Bedford, MA, USA). After blocking with TBST (150 mM NaCl, 10 mM Tris–HCl, and 0.05% v/v Tween 20, pH 7.5) containing 5% bovine serum albumin for 1 h at room temperature, the PVDF membranes were incubated with the biotinylated lectins (Vector Laboratories, Burlingame, CA, USA) from *Lens culinaris* (LCA), *Canavalia ensiformis* (ConA), *Lycopersicon esculentum* (LEL) and *Sambucus nigra* (SNA) for 2 h at room temperature. The membranes were subsequently incubated with horseradish peroxidase streptavidin (Vector Laboratories) for 30 min and detected with an ECL assay kit.

### Statistical analyses

The means of continuous data were compared using Student’s *t* test with SPSS software (version 16.0); *p* < 0.05 was considered statistically significant. The results shown in the figures are expressed as the mean ± SD.

The Feature Extraction tool (version 10.7.1.1, Agilent Technologies) was used to analyze the array images to obtain raw data. GeneSpring (version 13.1, Agilent Technologies) was employed to complete the basic analysis with the raw data. The raw data were initially normalized with the quantile algorithm. Differentially expressed genes were then identified by analyzing the fold change as well as the *P* value calculated with the *t* test. The threshold set for up- and down-regulated genes was a fold change of ≥1.5 with a *P* value ≤0.05 and a normalized signal intensity of either cell >8. To obtain information on glycogenes, we used the keywords “glyco”, “:glycan”, “gluc”, “gal”, “mgat”, “st6”, “st3”, “gnt”, “galnt”, “fuc”, “mannose”, and “alg” and then eliminated duplicate and false results, and focused on the glycosyltransferases that synthesize different structures.

## Results

### Viability of A2780 and A2780-cp cells when treated with cisplatin

**Fig. 1:**
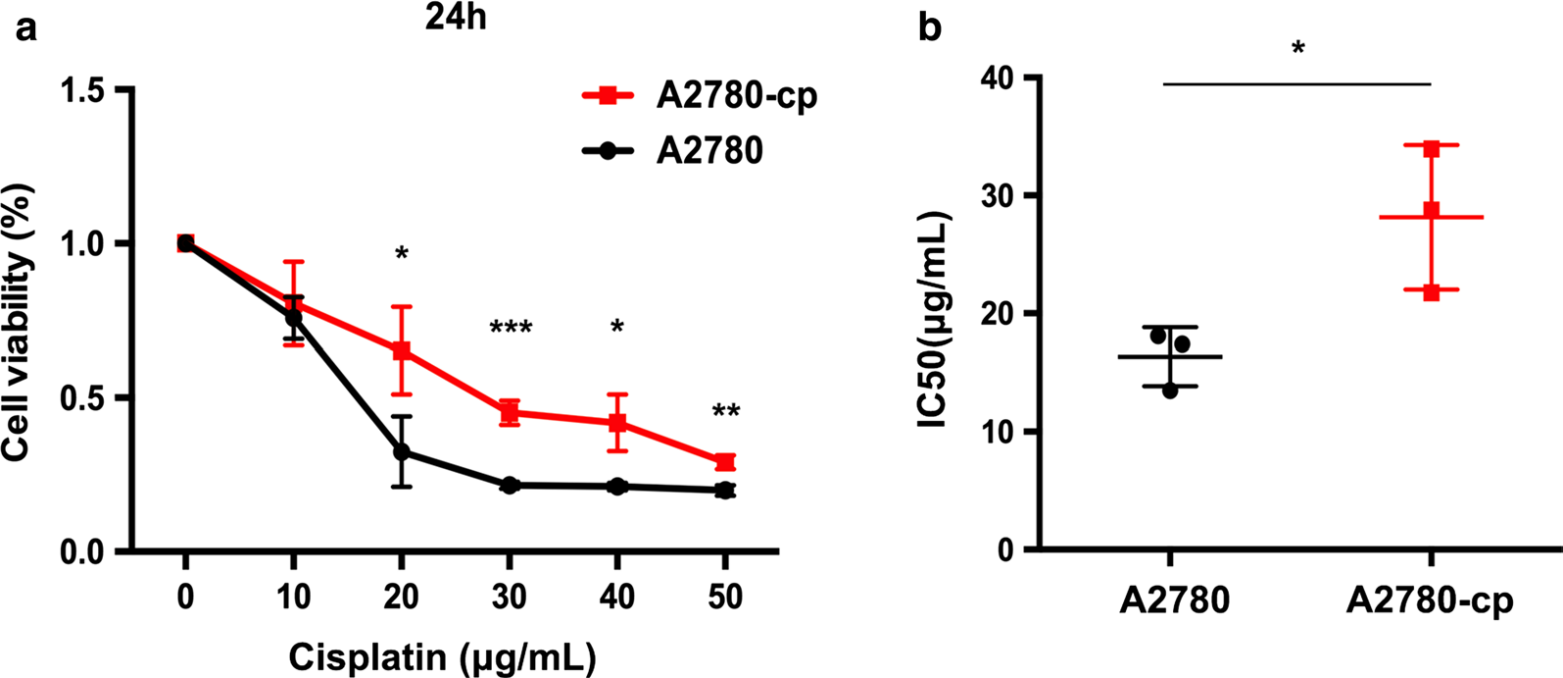
Viability of A2780 and A2780-cp cells after cisplatin incubation for 24 h determined via the CCK8 assay. **a** CCK8 analysis of A2780 and A2780-cp cells, *error bar*: standard deviation (repeat for three times); *p < 0.05; **p < 0.01; ***p < 0.001, Student’s *t* test; and **b** A2780-cp cells exhibited a significantly higher IC50

A CCK8 assay was conducted to validate the drug resistance of the A2780-cp cells as shown in Fig. 1. The viability of the A2780-cp cells was significantly higher than that of the A2780 cells. When the concentration was above 20 μg/ml, the viability of the A2780 cells dropped to <50%, whereas that of the A2780-cp cells remained higher than 50%. The IC50 of the A2780-cp cells was significantly higher than that of the A2780 cells (28.2 vs. 16.3 μg/ml, respectively).

### Lectin array analysis of A2780 cells and A2780-cp cells

**Fig. 2:**
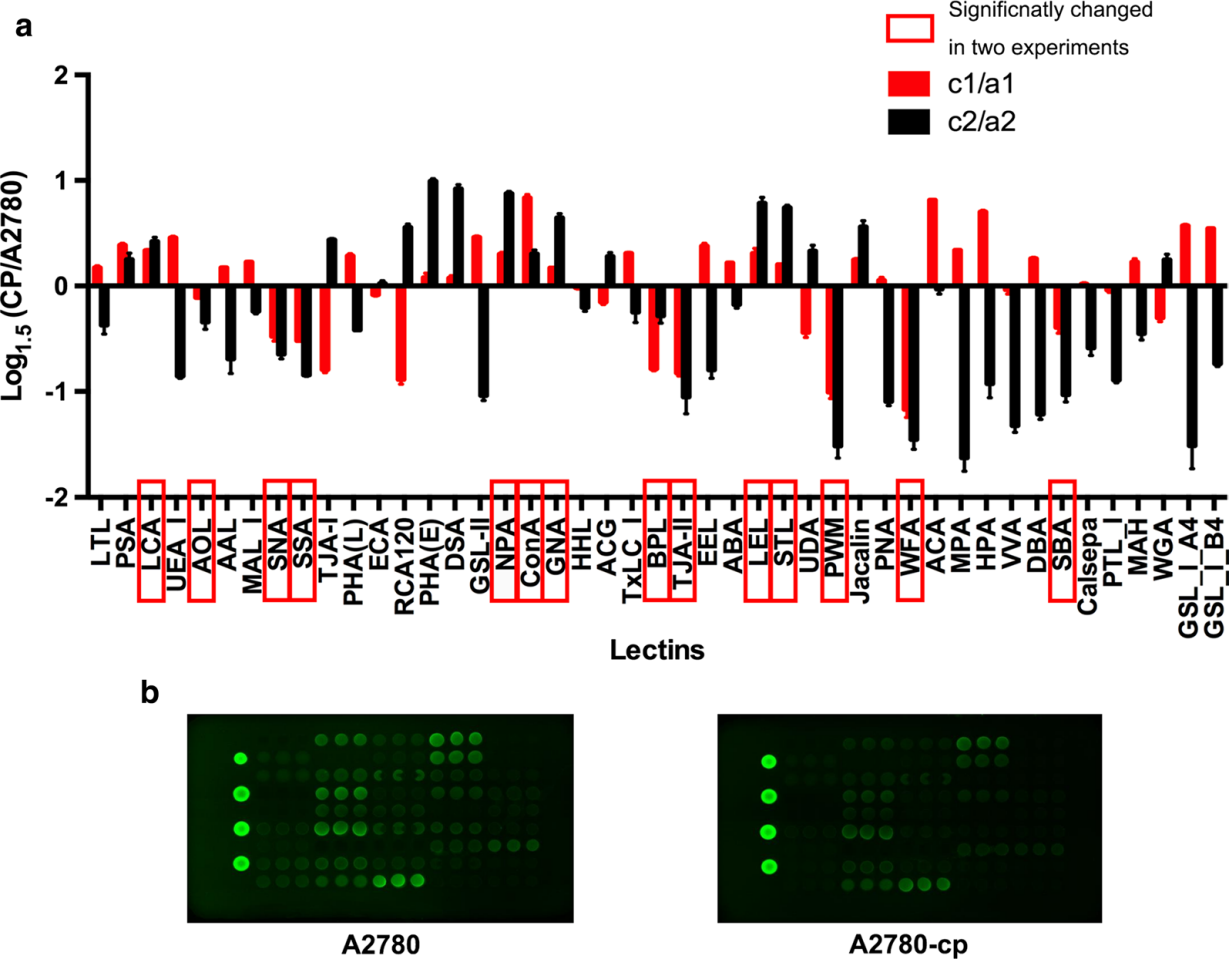
Lectin array analysis of A2780 cells and A2780-cp cells. **a** Relative intensity of 45 lectins in A2780 cells compared with that in A2780-cp cells in two experiments. Lectins that significantly changed consistently in two lectin array experiments were considered as significantly changed ones and are indicated with *red rectangles*. To show the results visually, the logarithm of the relative intensities (A2780-cp/A2780) to base 1.5 was calculated, and results of the first experiment are shown in the *red columns* and the second experiment were shown in the *black columns*. The *upper ones* were increased in A2780-cp cells, while the *lower lectins* were decreased. **b** Fluorescence intensities of A2780 cells (*left*) and A2780-cp cells (*right*), lectins in the array from *up to down* and then *left to right* are numbered from 1 to 45 in the Additional file 1

 A lectin array of 45 lectins was used to obtain more comprehensive information about the global glycosylation of A2780-cp cells for *N*-glycans and *O*-glycans and for the structures of other glycoconjugates, such as glycosphingolipids and proteoglycans. The results are shown in Fig. 2 and Table 1. Fourteen lectins exhibited significantly and consistently changes in the two independent experiments.

**Table 1:** Variations in glycans determined through lectin microarray analysis of the A2780 cells and A2780-cp cells

No.	Lectin	Preferred glycan structure (terminal epitope)	A2780-1 ± SD	CP-1 ± SD	C1/A1	A2780-2 ± SD	CP-2 ± SD	C2/A2
1	LTL	Fuc α1-3(Gal β1-4)GlcNAc (Le^x^), Fuc α1-2(Gal β1-4)GlcNAc	0.58 ± 0.02	0.62 ± 0.01	1.07**	0.47 ± 0.02	0.41 ± 0.02	0.86*
2	PSA	Fuc α1-6GlcNAc, α-D-Glc, α-D-Man	0.90 ± 0.03	1.06 ± 0.02	1.17***	0.94 ± 0.04	1.05 ± 0.07	1.11
3	LCA^a^	Fuc α1-6GlcNAc, α-D-Glc, α-D-Man	0.86 ± 0.04	0.99 ± 0.03	1.15***	1.02 ± 0.02	1.21 ± 0.08	1.19**
4	UEA-I	Fuc α1-2(Gal β1-4)GlcNAc	0.43 ± 0.02	0.52 ± 0.02	1.20***	0.48 ± 0.01	0.34 ± 0.00	0.71***
5	AOL^a^	Fuc α1-6GlcNAc (core Fuc), Fuc α1-2(Gal β1-4)GlcNAc	0.49 ± 0.02	0.47 ± 0.01	0.96***	0.52 ± 0.03	0.46 ± 0.02	0.87*
6	AAL	Fuc α1-6GlcNAc (core Fuc), Fuc α1-3(Gal β1-4)GlcNAc (Le^x^)	0.80 ± 0.03	0.86 ± 0.04	1.07***	1.04 ± 0.09	0.78 ± 0.02	0.76*
7	MAL	Sia α2-3Gal β1-4GlcNAc	0.69 ± 0.01	0.75 ± 0.01	1.10***	0.61 ± 0.01	0.56 ± 0.01	0.91**
8	SNA^a^	Sia α2-6Gal/GalNAc	1.01 ± 0.07	0.83 ± 0.03	0.82**	0.94 ± 0.02	0.72 ± 0.04	0.77***
9	SSA^a^	Sia α2-6Gal/GalNAc	0.95 ± 0.02	0.77 ± 0.02	0.81***	0.94 ± 0.01	0.67 ± 0.01	0.71***
10	TJA-I	Sia α2-6Gal/GalNAc	1.98 ± 0.10	1.44 ± 0.10	0.73***	1.16 ± 0.04	1.38 ± 0.06	1.19***
11	PHA-L	Tri/Tetra-antennary complex-type *N*-glycan	0.62 ± 0.02	0.69 ± 0.01	1.12**	0.58 ± 0.02	0.49 ± 0.01	0.85***
12	ECA	Gal β1-4GlcNAc	1.26 ± 0.04	1.22 ± 0.05	0.97*	0.98 ± 0.03	0.99 ± 0.05	1.01
13	RCA120	Gal β1-4GlcNAc	2.36 ± 0.09	1.65 ± 0.12	0.70***	1.97 ± 0.11	2.47 ± 0.23	1.25***
14	PHA-E	Complex-type *N*-glycans with outer Gal and bisecting GlcNAc	1.11 ± 0.02	1.15 ± 0.03	1.03	1.04 ± 0.05	1.55 ± 0.05	1.50***
15	DSA	(GlcNAc β1-4)_n_, Gal β1-4GlcNAc, Tri/Tetra-antennary *N*-glycans	2.57 ± 0.03	2.65 ± 0.09	1.03	1.58 ± 0.06	2.29 ± 0.14	1.45***
16	GSL-II	Agalactosylated tri/tetra antennary glycans, GlcNAc	0.43 ± 0.02	0.52 ± 0.02	1.20***	0.48 ± 0.02	0.31 ± 0.01	0.66***
17	NPA^a^	High Man, Man α1-6Man	1.30 ± 0.02	1.47 ± 0.05	1.13***	1.29 ± 0.08	1.85 ± 0.14	1.43***
18	ConA^a^	High Man, Man α1-6(Man α1-3)Man (inhibited by presence of bisecting GlcNAc)	1.46 ± 0.08	2.05 ± 0.03	1.40***	1.93 ± 0.16	2.19 ± 0.15	1.13**
19	GNA^a^	High Man, Man α1-3Man	1.05 ± 0.04	1.12 ± 0.05	1.07***	0.97 ± 0.04	1.27 ± 0.11	1.30***
20	HHL	High Man, Man α1-3Man, Man α1-6Man	0.75 ± 0.02	0.75 ± 0.02	0.99	0.82 ± 0.02	0.76 ± 0.04	0.92*
21	ACG	Sia α2-3Gal β1-4GlcNAc	1.45 ± 0.04	1.37 ± 0.07	0.94	1.55 ± 0.06	1.74 ± 0.13	1.12**
22	TxLC-I	Man_3_ core, bi- and tri-antennary complex-type *N*-glycan, GalNAc	0.64 ± 0.02	0.72 ± 0.02	1.13**	0.75 ± 0.02	0.68 ± 0.04	0.90
23	BPL^a^	Gal β1-3GalNAc (α-Thr/Ser (T)), GalNAc	0.82 ± 0.02	0.60 ± 0.01	0.73***	0.58 ± 0.01	0.52 ± 0.03	0.89*
24	TJA-II^a^	Fuc α1-2Gal β1-4, GalNAcβ1-4 groups at their nonreducing terminals	1.24 ± 0.03	0.89 ± 0.01	0.72***	1.19 ± 0.06	0.78 ± 0.09	0.65**
25	EEL	Gal α1-3Gal β1-4GlcNAc, Fuc α1-2(Gal α1-3)Galβ1-4GlcNAc	0.44 ± 0.02	0.52 ± 0.02	1.17***	0.41 ± 0.02	0.30 ± 0.02	0.72**
26	ABA	Gal β1-3GalNAc (α-Thr/Ser (T)), GlcNAc, sialyl-T	0.86 ± 0.01	0.94 ± 0.01	1.09***	1.01 ± 0.04	0.94 ± 0.05	0.93*
27	LEL^a^	(GlcNAc β1-4)_n_, (Gal β1-4GlcNAc)_n_ (polyLacNAc)	3.22 ± 0.28	3.65 ± 0.14	1.13*	2.37 ± 0.08	3.26 ± 0.18	1.37**
28	STL^a^	(GlcNAc)_n_, (GlcNAc β1-4MurNAc)_n_ (peptidoglycan backbone)	2.72 ± 0.21	2.95 ± 0.22	1.09***	1.48 ± 0.06	2.00 ± 0.13	1.35***
29	UDA	GlcNAc β1-4GlcNAc, Mixture of Man_5_ to Man_9_	1.97 ± 0.22	1.65 ± 0.13	0.84**	1.72 ± 0.06	1.97 ± 0.18	1.14*
30	PWM^a^	(GlcNAc α1-4)_n_	0.78 ± 0.08	0.52 ± 0.03	0.67***	0.71 ± 0.03	0.39 ± 0.04	0.54***
31	Jacalin	Gal β1-3GalNAc (α-Thr/Ser (T)), GalNAc (α-Thr/Ser (Tn))	1.02 ± 0.07	1.13 ± 0.08	1.11	1.50 ± 0.05	1.89 ± 0.18	1.25**
32	PNA	Gal β1-3GalNAc (α-Thr/Ser (T))	0.40 ± 0.02	0.41 ± 0.03	1.02	0.62 ± 0.02	0.40 ± 0.02	0.64***
33	WFA^a^	Terminal GalNAc (e.g. GalNAcβ1-4GlcNAc), Galβ1-3(-6)GalNAc	0.94 ± 0.08	0.59 ± 0.02	0.62***	0.79 ± 0.03	0.44 ± 0.02	0.55***
34	ACA	Gal β1-3GalNAc (α-Thr/Ser (T))	0.87 ± 0.06	1.21 ± 0.07	1.39***	1.26 ± 0.05	1.25 ± 0.04	0.99
35	MPA	Gal β1-3GalNAc (α-Thr/Ser (T)), GalNAc (α-Thr/Ser (Tn))	0.61 ± 0.06	0.70 ± 0.06	1.15***	1.12 ± 0.04	0.58 ± 0.03	0.52***
36	HPA	α-Linked terminal GalNAc	0.42 ± 0.04	0.56 ± 0.04	1.33***	0.74 ± 0.02	0.51 ± 0.05	0.69**
37	VVA	α-Linked terminal GalNAc (α-Thr/Ser (Tn), GalNAc α1-3Gal	0.46 ± 0.01	0.45 ± 0.02	0.99	0.87 ± 0.05	0.51 ± 0.02	0.58***
38	DBA	GalNAc α1-3GalNAc (Blood group A), GalNAc α1-3GalNAc	0.40 ± 0.01	0.44 ± 0.01	1.11***	0.61 ± 0.05	0.37 ± 0.02	0.61***
39	SBA^a^	α or β-linked terminal GalNAc, GalNAc α1-3Gal	0.49 ± 0.00	0.42 ± 0.02	0.85**	0.80 ± 0.09	0.53 ± 0.05	0.66***
40	Calsepa	High Man (Man_2-6_), *N*-glycans including bisecting GlcNAc	0.59 ± 0.01	0.59 ± 0.00	1.01	1.15 ± 0.11	0.90 ± 0.07	0.79**
41	PTL-I	α-Linked terminal GalNAc	0.52 ± 0.01	0.51 ± 0.01	0.99	0.75 ± 0.05	0.52 ± 0.04	0.70***
42	MAH	Sia α2-3Gal β1-3(Sia α2-6) GalNAc	0.48 ± 0.01	0.52 ± 0.02	1.10*	0.45 ± 0.00	0.37 ± 0.02	0.83**
43	WGA	(GlcNAcβ1-4)_n_, NeuAc, multivalent Sia	1.26 ± 0.01	1.12 ± 0.04	0.89**	1.26 ± 0.03	1.39 ± 0.07	1.11*
44	GSL-IA_4_	α-GalNAc (α-Thr/Ser (Tn))	0.39 ± 0.01	0.49 ± 0.01	1.26***	0.69 ± 0.07	0.37 ± 0.02	0.54**
45	GSL-IB_4_	α-Gal	0.39 ± 0.01	0.49 ± 0.00	1.25***	0.87 ± 0.05	0.65 ± 0.03	0.74***

^a^Significant changes in the two experiments. * *p* < 0.05; ** *p* < 0.01; *** *p* < 0.001

The intensity of the LCA lectin, which recognizes fucose α1-6 *N*-acetylgalactosamine (Fuc α1-6 GlcNAc) structures, was significantly higher in A2780-cp cells, whereas the intensity of the *Aspergillus oryzae* lectin (AOL), which recognizes Fuc α1-6GlcNAc and Fuc α1-2(gal β1-4) GlcNAc, was significantly lower in the A2780-cp cells. *Pisum sativum* (PSA) which could recognize Fuc α1-6GlcNAc was increased in A2780-cp cells in two lectin array experiments, while lectin *Trichosanthes japonica* (TJA-II) lectin, which could recognize Fuc α1-2(Gal β1-4) GlcNAc was significantly decreased in A2780-cp cells. These findings were further verified by the elevated transcription level of fucosyltransferase-8 (Fut8) which catalyzed Fuc α1-6GlcNAc structure, and the reduced level of Fut1which catalyzed Fuc α1-2Gal β1-4 structure. These results together indicated that Fuc α1-6GlcNAc was overexpressed in the A2780-cp cells, whereas Fuc α1-2(Gal β1-4) GlcNAc expression was decreased.

Among the sialic acid (Sia) binders, the A2780-cp cells showed a significantly decreased intensity of SNA and SSA (*Sambucus sieboldiana*), indicating the lower expression of α2-6-linked Sia.

Moreover, A2780-cp cells showed stronger intensities of the lectins NPA (*Narcissus pseudonarcissus*), ConA (*C. ensiformis*), and GNA (*Galanthus nivalis*), which are high mannose-type glycan binders. This finding demonstrated that a large amount of less mature glycans are synthesized in the A2780-cp cells.

A2780-cp cells also exhibited significantly weaker intensities of lectins that recognize terminal Gal or GalNAc, such as BPL (*Bauhinia purpurea alba*), TJA-II (*T. japonica*), WFA (*Wisteria floribunda*) and SBA (*Glycine max*).

The LEL intensity was higher in A2780-cp cells, demonstrating that the expression of the poly *N*-acetyllactosamine (poly-LacNAc: Gal β1-4 GlcNAc)_n_ structure was elevated in A2780-cp cells. In addition, STL (*Solanum tuberosum lectin*) showed a stronger signal intensity, while PWM (*Phytolacca americana*) showed a weaker signal intensity in the A2780-cp cells.

Overall, 5 lectins exhibited a significantly stronger intensity in A2780-cp cells, indicating that the core Fuc, high mannose, and poly LacNAc lectins were overexpressed in the A2780-cp cells. Additionally, the signals for 9 lectins were significantly lower, revealing that the expression of the α2-6-linked Sia and terminal Gal or GalNAc lectins was reduced in A2780-cp cells.

### Variation of glycogene expression in A2780-cp cells

**Fig. 3:**
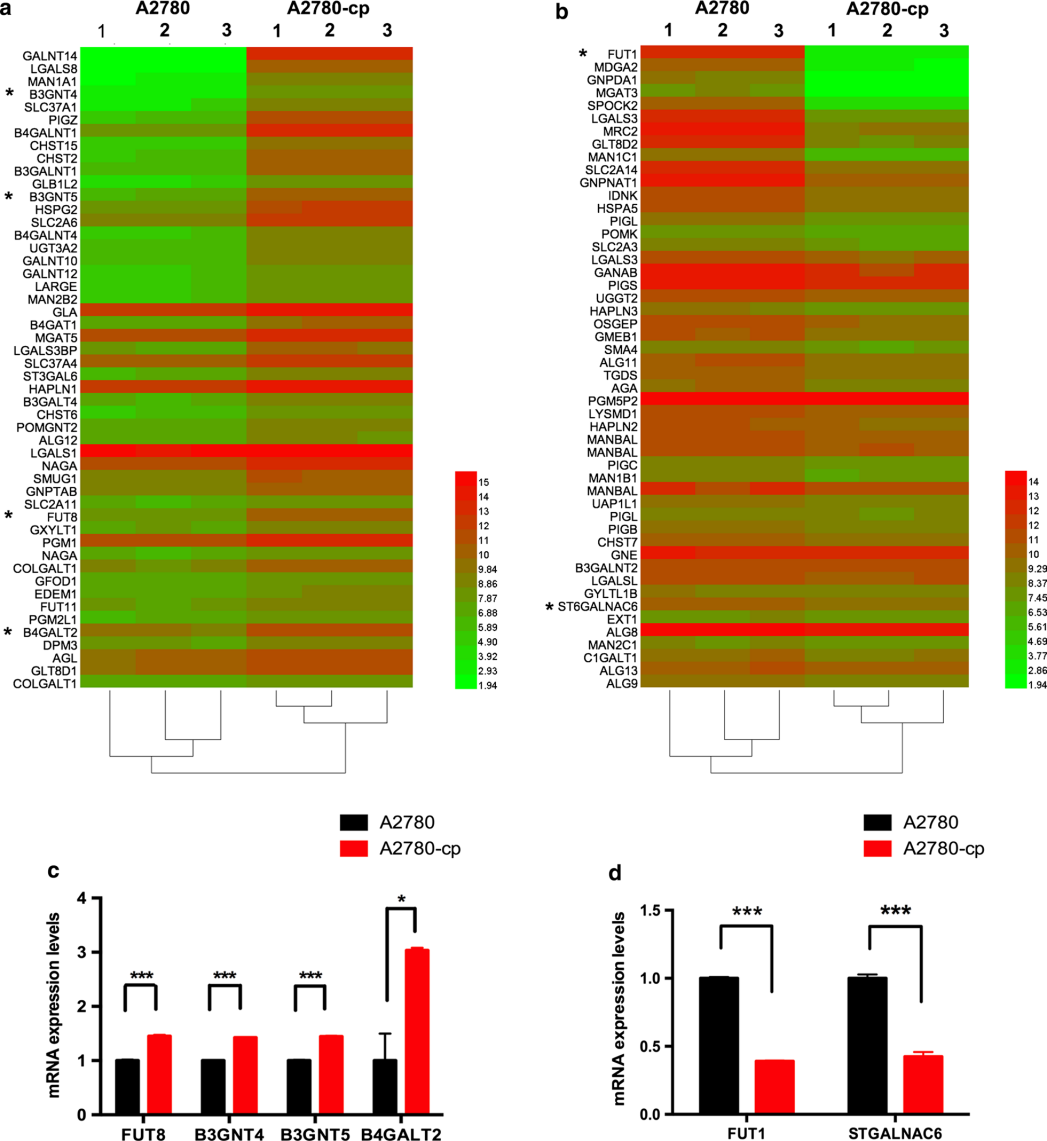
Heatmap of the altered glycan gene expression. **a** The 50 genes exhibiting the greatest increases are shown in the heat map. **b** The 50 genes exhibiting the greatest decreases are shown in the heatmap. *Genes showing significant changes that correlated well with the lectin array results. **c**, **d** Real-time PCR validation of expression array results

To obtain the glycogene expression profile of A2780-cp cells, we performed a gene expression microarray analysis, selected the related glycogenes according to the rules mentioned in the “Methods” section and visualized the data using hierarchical clustering (as shown in Fig. 3; Additional file 3). Genes that significantly changed over 1.5 folds were selected, the top increased 50 and decreased 50 genes were shown in the heatmap (Fig. 3). The results of the gene array analysis were consistent with the lectin results. The gene encoding FUT8, which catalyzes the synthesis of the core fucosylated (Fuc α1-6 GlcNAc) glycan, was significantly upregulated in the A2780-cp cells, whereas the expression of the gene encoding FUT1, which catalyzes the synthesis of Fuc α1-2(gal β1-4) GlcNAc, was decreased. Moreover, the results showed that the expression of a series of genes encoding proteins involved in poly-LacNAc synthesis, including UDP-GlcNAc:βGal β-1,3-N-acetylglucosaminyltransferase 4 (B3Gnt4), B3Gnt5, β-1,4-galactosyltransferase 2 (B4GalT2) were significantly increased in the A2780-cp cells. Additionally, the gene for ST6 *N*-acetylgalactosaminide α-2,6-sialyltransferase 6 (ST6GalNAc6), which adds Sia to a GalNAc through an α2,6 linkage, was significantly down-regulated in the A2780-cp cell line. These results validated lectin array results, the core fucosylation and poly-lacNAc structures were increased and α2-6-linked Sia were decreased in A2780-cp. Expression of these genes were verified via realtime PCR.

Other glycogenes encoding enzymes that perform the first step in the synthesis of O-glycan, such as *galnt14*, *galnt10* and *galnt12*, are overexpressed in the A2780-cp cells. The gene expression of the Mgat5, which is involved in the synthesis of multi-branched complex N-glycan, was increased in the A2780-cp cells, whereas that of Mgat3, which is involved in synthesizing the bisecting complex N-glycan, was decreased. The expression of the genes encoding ST3 beta-galactoside α-2,3-sialyltransferase 6 (St3gal6), St3gal2, and St3gal4, which synthesize the Sia2,3-gal structure, was elevated in the A2780-cp cells.

### Lectin blot to verify the results changed consistently in both arrays analysis

**Fig. 4:**
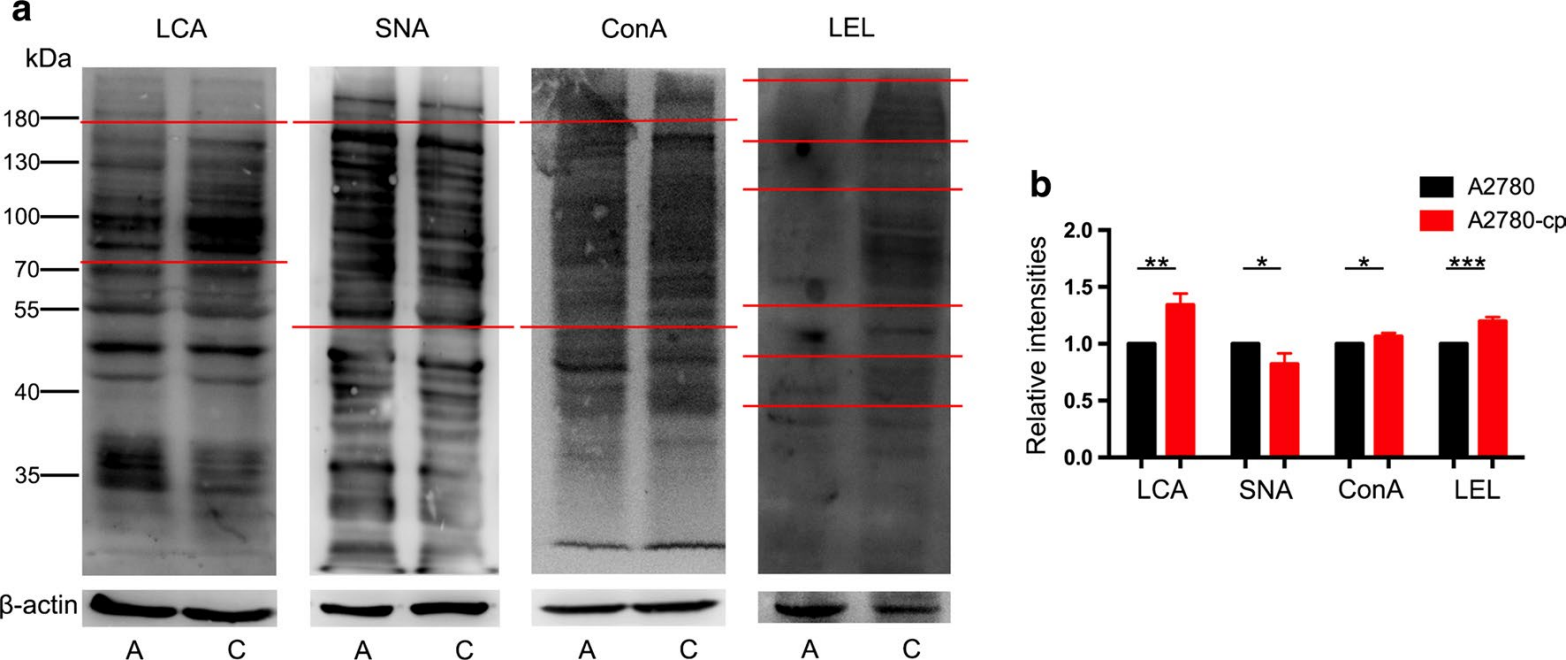
Lectin blot analysis of A2780 and A2780-cp cells. **a** LCA, SNA, Con A, LEL were used for lectin blot analysis. **b** Relative intensities of lectin blot. *A* A2780 cells, *C* A2780-cp cells

We integrated the results of lectin array and gene expression array analysis, three kinds of glycan structures (core fucosylation, α2,6 linked sia, poly-LacNAc structures) were consistently significantly changed in A2780-cp cells. Additional, high mannose didn’t have a corresponding significant changed gene in gene expression array, but all the lectins recognized high mannose structures (NPA, ConA, GNA) were consistently increased in A2780-cp. To further validate the lectins changed consistently in both arrays, we performed lectin blots using three lectins (LCA, SNA and LEL). We also verified the high mannose structure using ConA. The A2780-cp cell line showed significantly increased intensities of LCA, ConA and LEL, but a significantly decreased intensity for SNA (Fig. 4). These results were consistent with the results of two arrays. The following results were observed for the A2780-cp cells: for LCA, glycoproteins from 70 to 180 kDa exhibited a higher intensity; for ConA, glycoproteins from 50 to 180 kDa exhibited a higher intensity; for LEL, glycoproteins from 50 to 110 kDa, 40 to 50 kDa and above 180 kDa exhibited a higher intensity; and for SNA, glycoproteins from 50 to 180 kDa exhibited a lower intensity.

## Discussion

Relapse and drug resistance after the initial treatment are the main challenges in treating ovarian cancer [19]. Many studies have found that glycosylation exhibits an intimate connection with the mechanism underlying drug resistance [11]. However, few glycomic analyses have been performed on drug resistant cells, and prior glycomic analyses have focused on serum samples. Cell lines are more stable than serum samples and can facilitate research on resistance mechanisms and may provide diagnostic, prognostic, and therapeutic biomarkers [21]. In the present study, we profiled the glycomics of the coupled cell lines A2780 and A2780-cp using a lectin array, which represents a time saving (one-day operation) approach with convenient sample preparation (without derivatization), and it only requires only small numbers of cells (lower limit of 1000 cells). To obtain additional information on the regulation of gene expression, we performed a gene expression array analysis to facilitate further investigations of this drug-resistant cell model.

Our results based on the combination of lectin and gene arrays indicated that the expression of the core fucosylation and poly-LacNAc structures was increased, while the expression of the Fuc α1-2(gal β1-4) GlcNAc and α2-6-linked Sia structures was decreased in A2780-cp cells. In prior *N*-glycomic analyses of ovarian cancer serum samples, Alley and colleagues found that tri- and tetra-branched structures with varying degrees of sialylation and fucosylation were increased and “bisecting” glycans were decreased in late-stage recurrent ovarian cancer patients [17]. Our gene array analysis presented consistent results (shown in Fig. 4) and revealed that the expression of mgat5, which catalyzes the formation of a multi-branched structure, was increased, while the expression of mgat3, forming bisecting complex *N*-glycan, was decreased. However, our lectin results did not reveal a consistent significant change in L-PHA and E-PHA.

A number of studies have shown that fucosylation and sialylation are involved in drug resistance. Cheng et al. [22] reported that Fut4, Fut6, and Fut8 mediate the multidrug-resistant phenotype in hepatocellular cancer via the PI3 K/Akt signaling pathway. ST6GAL1 has the reversed effect on the activity of PI3 K/Akt signaling and regulates the expression of P-gp and MRP1, which is related to multidrug resistance [13]. ST6Gal1 also blocks cisplatin-induced cell death by reducing the activation of caspase 3 in ovarian cancer [23]. Our results showed that Fut8 was significantly increased, whereas Fut1 and ST6GalNAc6 were significantly decreased in A2780-cp cells. Accordingly, the LCA, AOL and SNA lectins showed significant changes in the lectin array analysis.

In addition, poly-LacNAc was elevated in A2780-cp cells. Poly-LacNAc is a common structure that can be observed in complex *N*-glycans, *O*-glycans, glycosphingolipids, and certain proteoglycans, and it should be considered in future studies. This structure is recognized by galectins, which are involved in cell apoptosis and drug resistance in carcinomas [24]. Galectin-1-induced autophagy could facilitate cisplatin resistance in hepatocellular cancer [25]. Galectin-3 inhibition contributes to calpain activation and sensitizes prostate cancer cells to cisplatin [26]. Thus, poly-LacNAc might participate in cisplatin resistance via galectin and a downstream signaling pathway, such as PI3 K/Akt.

These results have some clinical significance. These different expressed glycans maybe potential biomarkers which could discriminate cisplatin resistant cells from sensitive cells, it would predict patient’s response to cisplatin treatment before chemotherapy, and has potential in determining the most suitable chemotherapy drug prior to treatment. Additionally, glycan can be used with traditional chemotherapy for precisely targeting.

The present study provides glycomic information on the two studied cell lines, and further studies should involve perform glycomics analyses of cisplatin-resistant cancer tissues and focus on the mechanisms underlying the involvement of glycans in drug resistance.

## Conclusions

The cisplatin-resistant cell line A2780-cp exhibited higher expression of core fucosylation, poly-LacNAc and high mannose structures and lower expression of Fuc α1-2(gal β1-4) GlcNAc and α2-6-linked Sia structures. These glycans might represent potential biomarkers and could be involved in the mechanism of drug resistance in ovarian cancer.

## Additional files

**Additional file 1.** Lectins in lectin array.

**Additional file 2.** Primer sequences.

**Additional file 3.** Significantly changed glycan-related genes in gene expression array.

## Abbreviations

PI3K: phosphoinositide 3 kinase
LCA: *Lens culinaris*
ConA: *Canavalia ensiformis*
LEL: *Lycopersicon esculentum*
SNA: *Sambucus nigra*
AOL: *Aspergillus oryzae* lectin
Fuc: fucose
Gal: galactose
GalNAc: *N*-acetylgalactosamine
Fut: fucosyltransferase
Sia: sialic acids
SSA: *Sambucus sieboldiana*
NPA: *Narcissus pseudonarcissus*
GNA: *Galanthus nivalis*
BPL: *Bauhinia purpurea alba*
TJA-II: *Trichosanthes japonica*
WFA: *Wisteria floribunda*
SBA: *Glycine max*
LacNAc: *N*-acetyllactosamine
STL: *Solanum tuberosum lectin*
PWM: *Phytolacca americana*
B3GnT: UDP-GlcNAc: βGal β-1,3-*N*-acetylglucosaminyltransferase
B4GalT: β-1,4-Galactosyltransferase
ST6GalNAc6: ST6 *N*-acetylgalactosaminide α-2,6-sialyltransferase 6
ST3Gal6: ST3 beta-galactoside α-2,3-sialyltransferase 6
SDS-PAGE: sodium dodecylsulfate-polyacrylamide gel electrophoresis
PVDF: polyvinylidene difluoride
